# Dataset on hybrid car adoption behaviour and sustainability perceptions in Bangladesh

**DOI:** 10.1016/j.dib.2026.113031

**Published:** 2026-06-26

**Authors:** Md. Aslam Uddin, Mohammad Zahedul Alam, Md. Masudul Islam

**Affiliations:** a Bangladesh University of Business and Technology, Dhaka, Bangladesh; b Bangladesh University of Professionals, Dhaka, Bangladesh

**Keywords:** UTAUT2, EC, HBs, CCB, Technology acceptance, Emerging economy

## Abstract

This article presents a survey-based dataset examining hybrid car adoption behaviour and sustainability-related perceptions among consumers in Bangladesh. The dataset was developed to investigate factors influencing behavioural intention toward hybrid vehicle adoption using the Unified Theory of Acceptance and Use of Technology (UTAUT2) framework together with additional sustainability-oriented behavioural constructs. The survey instrument includes demographic variables and twelve latent constructs, namely Performance Expectancy (PE), Effort Expectancy (EE), Role of Salesperson (RS), Facilitating Conditions (FC), Social Influence (SI), Hedonic Motivation (HM), Price Value (PV), Health Benefit (HB), Environmental Concern (EC), Personal Innovativeness (PI), Consumer Citizenship Behaviour (CCB), and Behavioural Intention to Purchase (BIP). Data were collected through structured online and paper-based questionnaires administered among urban and semi-urban consumers familiar with hybrid vehicle technologies. After pre-processing and removal of incomplete responses, the final dataset consisted of 474 valid observations. Responses were measured using a five-point Likert scale and prepared for descriptive statistical analysis and structural equation modelling applications. The dataset includes raw responses, questionnaire instruments, variable coding schemes, and a complete codebook to facilitate reproducibility and secondary analysis. The data provide useful insight into sustainable mobility perceptions, green technology adoption behaviour, and environmentally conscious transportation preferences within an emerging economy context. Researchers may reuse the dataset for regression analysis, machine learning, clustering, consumer segmentation, and structural equation modelling, while policymakers and practitioners may utilize the findings to support sustainable transportation planning and green mobility policy development in developing countries.

Specifications Table

**Table:** 

Subject	Social Sciences
Specific subject area	Consumer behaviour and technology adoption focusing on hybrid car acceptance, sustainability attitudes, and Behavioural intentions in an emerging economy
Type of data	Table, Raw
Data collection	Data were collected via a structured questionnaire survey using 5-point Likert scales. Items were adapted from validated UTAUT2 and green consumption literature, with constructs such as EC, HBs, and CCB. The instrument was pretested and refined for clarity. Data were gathered through self-administered online and paper-based surveys from respondents familiar with or interested in hybrid cars. The survey was conducted between March 2025 to July 2025, and respondents took approximately 10–15 min to complete the questionnaire. Each respondent participated only once, and duplicate submissions were screened and removed during the pre-processing stage. Incomplete or inconsistent responses were excluded, and data were coded and cleaned in spreadsheet software. After pre-processing and exclusion of incomplete responses, 474 valid responses were retained from 510 collected questionnaires.
Data source location	Data were collected from respondents in Bangladesh, primarily in urban and semi-urban areas (Dhaka and surrounding regions) where awareness and exposure to hybrid vehicle technologies are comparatively higher due to greater economic activity, transportation demand, and access to technological information. The dataset is stored and managed at the authors’ institutional repository/affiliated university for academic research purposes.
Data accessibility	Repository name: Mendeley Data Data identification number: 10.17632/hbytbmttz7.4 Direct URL to data: https://data.mendeley.com/datasets/hbytbmttz7/4 Instructions for accessing these data: The dataset is publicly available on Mendeley Data and can be accessed via the provided DOI link. Users can open the dataset landing page, where all associated files including the Excel dataset and questionnaire are listed. The files can be viewed online or downloaded directly without restriction for academic and research purposes. No special permissions or registration are required to access the data. The dataset is structured to allow replication of statistical analyses using standard software such as SPSS, SmartPLS, R, or Python. The variable coding scheme and questionnaire mapping enable reproducibility of descriptive, reliability, and structural modelling analyses.
Related research article	Not applicable.

## 1 Value of the Data

• This dataset provides a comprehensive and structured representation of hybrid car adoption behaviour by integrating UTAUT2 constructs with extended behavioural factors, including EC, HBs, PI, and CCB. The inclusion of CCB offers additional insight into advocacy and post-adoption engagement, which is relatively underexplored in existing adoption datasets.
• The data represent one of the few publicly available datasets on sustainable transportation adoption from Bangladesh and the broader South Asian region. This makes it particularly valuable for researchers examining technology adoption in emerging economies, where socio-economic conditions, infrastructure constraints, and environmental awareness differ significantly from developed contexts.
• The dataset enables both behavioural Intention analysis and segmentation based on demographic variables such as income, education, and occupation, supporting deeper insights into heterogeneous consumer groups in developing markets.
• Researchers can reuse this dataset for a variety of analytical approaches, including structural equation modelling (SEM), regression analysis, classification and clustering in machine learning, and comparative cross-country studies. It is also suitable for validating and extending theoretical frameworks such as UTAUT2 in the context of green and sustainable technologies.
• Policymakers and practitioners can utilize the data to identify key drivers of hybrid car adoption, assess public readiness for sustainable mobility transitions, and design targeted interventions to promote environmentally friendly transportation solutions in resource-constrained settings.

## 2 Background

Hybrid vehicles have attracted increasing attention in Bangladesh due to rising fuel costs, growing environmental pollution, rapid urbanization, and increasing public awareness regarding sustainable transportation alternatives. Understanding consumer perceptions and behavioural intentions toward hybrid vehicle adoption is therefore important for promoting environmentally sustainable mobility transitions in developing countries. The study is grounded in established technology acceptance theories, particularly the Unified Theory of Acceptance and Use of Technology (UTAUT2) framework [1], which includes constructs such as PE, EE, SI, FC, HM, and PV. To provide broader behavioural and sustainability-related insight, the study additionally incorporated constructs associated with EC, HB, PI, RS, and CCB based on prior literature related to sustainable consumption, innovation adoption, and green technology acceptance [[2], [3], [4], [5], [6]]. The RS construct was incorporated as an additional standalone construct extending beyond the original UTAUT2 framework. The construct was included based on prior literature emphasizing the importance of interpersonal communication, salesperson influence, and consumer guidance during technology-related purchase decision processes. Its inclusion was considered particularly relevant within the context of Bangladesh and similar emerging economies, where consumers often rely on direct interpersonal interaction, trust-building communication, and salesperson-provided information when evaluating relatively unfamiliar or technologically advanced products such as hybrid vehicles. Therefore, the construct was expected to provide additional behavioural insight beyond the traditional UTAUT2 dimensions. A structured questionnaire was developed using validated measurement items adapted from existing studies, with minor contextual modifications introduced to improve suitability for hybrid car adoption research in Bangladesh. Responses were collected using a five-point Likert scale through a cross-sectional survey administered among respondents familiar with or interested in hybrid vehicles. The target population consisted of economically active adult consumers residing in urban and semi-urban regions of Bangladesh who possessed basic awareness of hybrid vehicle technologies and sustainable transportation concepts. Eligible respondents were required to be at least 18 years old and capable of independently completing the questionnaire in either online or paper-based format. The survey particularly focused on individuals with potential purchasing influence or exposure to automobile-related decision-making, including businesspersons, service holders, professionals, and educated consumers familiar with emerging transportation technologies. The dataset contains demographic variables together with multi-item construct indicators suitable for descriptive statistical analysis, structural equation modelling, machine learning applications, and comparative behavioural studies. This data article provides a comprehensive description of the dataset, including variable structure, coding scheme, measurement scales, pre-processing procedures, and reliability assessment to facilitate transparency, reproducibility, and secondary analysis. The dataset complements related research on electric and hybrid vehicle adoption [[2], [3], [4], [5], [6], [7], [8], [9]] by providing publicly accessible survey data from Bangladesh, thereby supporting future comparative studies, methodological validation, and sustainable transportation research in emerging economy contexts (Table 1).

**Table 1:** Variable dictionary and coding structure.

Variable Name	Brief Description	Construct	Scale Type	Coding
AGE	Respondent age group	Demographic	Categorical	1 = 20–30, 2 = 30–40, 3 = 40–50, 4=Above 50
EDUC	Education level	Demographic	Categorical	1=Below SSC, 2=SSC, 3=HSC, 4=Bachelor, 5=Masters, 6=MPhil/PhD
GEND	Gender	Demographic	Binary	1=Male, 2=Female
OCCUP	Occupation	Demographic	Categorical	1=Business, 2=Service, 3=Professional, 4=Others
INCOME	Monthly income (BDT)	Demographic	Categorical	1 ≤ 50k, 2 = 50–100k, 3 = 100–150k, 4 = 150–200k, 5 = 200–250k, 6=>250k
PE1	Hybrid car is useful in daily life	PE	Likert	1–5
PE2	Improves achievement of important tasks	PE	Likert	1–5
PE3	Helps accomplish tasks quickly	PE	Likert	1–5
PE4	Reduces maintenance cost	PE	Likert	1–5
PE5	Improves standard of living	PE	Likert	1–5
EE 1	Easy to learn hybrid car usage	EE	Likert	1–5
EE 2	Interaction is clear and understandable	EE	Likert	1–5
EE 3	Easy to drive hybrid car	EE	Likert	1–5
EE 4	Easy to become skilful	EE	Likert	1–5
RS1	Salesperson behaviour influences purchase	RS	Likert	1–5
RS2	Salesperson attraction influences purchase	RS	Likert	1–5
RS3	Salesperson provides useful information	RS	Likert	1–5
RS4	Positive communication encourages purchase	RS	Likert	1–5
RS5	Salesperson plays vital role	RS	Likert	1–5
FC1	Availability of necessary resources	FC	Likert	1–5
FC2	Availability of knowledge	FC	Likert	1–5
FC3	Compatibility with other technologies	FC	Likert	1–5
FC4	Availability of help/support	FC	Likert	1–5
FC5	Compatibility with energy-saving preferences	FC	Likert	1–5
SI1	Important people think I should use it	SI	Likert	1–5
SI2	Influential people encourage use	SI	Likert	1–5
SI3	Valued opinions favor usage	SI	Likert	1–5
SI4	Family/friends support usage	SI	Likert	1–5
SI5	Trend influence on usage	SI	Likert	1–5
HM1	Driving is pleasing	HM	Likert	1–5
HM2	Using is enjoyable	HM	Likert	1–5
HM3	Using is entertaining	HM	Likert	1–5
PV1	Reasonably priced	PV	Likert	1–5
PV2	Price compared to alternatives is reasonable	PV	Likert	1–5
PV3	Good value for money	PV	Likert	1–5
PV4	Worth current price	PV	Likert	1–5
HB1	Health is important to me	HB	Likert	1–5
HB2	Good health matters	HB	Likert	1–5
HB3	I think about my health	HB	Likert	1–5
HB4	Concern about healthy car usage	HB	Likert	1–5
HB5	Concern about health consequences	HB	Likert	1–5
EC1	Hybrid car is environmentally friendly	EC	Likert	1–5
EC2	Concern about environmental problems	EC	Likert	1–5
EC3	Concern about energy conservation	EC	Likert	1–5
EC4	Environmental attitude influences purchase	EC	Likert	1–5
PI1	Others seek my advice on technology	PI	Likert	1–5
PI2	Others learn faster than me	PI	Likert	1–5
PI3	Early adopter of new technologies	PI	Likert	1–5
PI4	Can learn tech independently	PI	Likert	1–5
PI5	Keep up with new technology	PI	Likert	1–5
PI6	Open to learning new technology	PI	Likert	1–5
CCB1	Say positive things to others	CCB	Likert	1–5
CCB2	Give constructive suggestions	CCB	Likert	1–5
CCB3	Share ideas for improvement	CCB	Likert	1–5
CCB4	Report problems for improvement	CCB	Likert	1–5
CCB5	Recommend to others	CCB	Likert	1–5
CCB6	Encourage others to experience	CCB	Likert	1–5
BIP1	Intend to continue using	BIP	Likert	1–5
BIP2	Will try to use regularly	BIP	Likert	1–5
BIP3	Plan frequent usage	BIP	Likert	1–5

## 3 Data Description

**Fig. 1:**
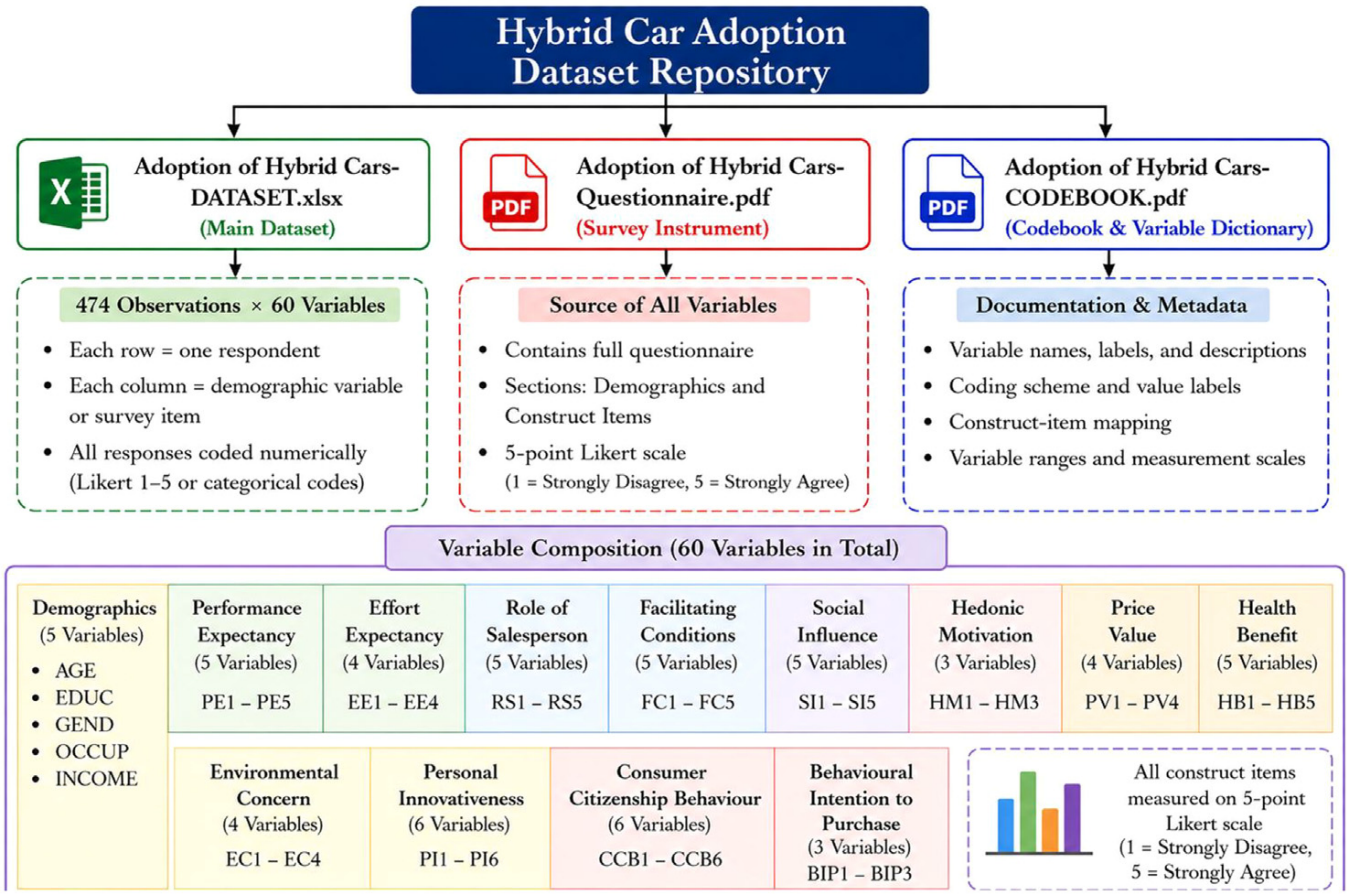
Dataset organization and variable composition.

The dataset repository [10] consists of 474 valid responses collected from consumers in Bangladesh to investigate behavioural intention and adoption-related perceptions toward hybrid cars. The Hybrid Car Adoption dataset repository is organized into three primary components: the main dataset file, the survey questionnaire, and the codebook with variable dictionary. The main dataset file (Adoption of Hybrid Cars-DATASET.xlsx) contains 474 valid respondent observations and 60 variables, where each row represents an individual respondent and each column corresponds to either a demographic variable or a construct measurement item. All responses are numerically coded using categorical labels and a five-point Likert scale. The survey questionnaire file (Adoption of Hybrid Cars-Questionnaire.pdf) provides the complete English-language survey instrument, including demographic questions and construct measurement items used during data collection. The codebook file (Adoption of Hybrid Cars-CODEBOOK.pdf) contains detailed metadata, variable descriptions, coding schemes, construct-item mappings, measurement scales, and explanations necessary for interpreting the dataset. The repository structure enables straightforward reuse of the dataset in statistical and machine learning software such as SPSS, SmartPLS, R, Python, and STATA for reproducibility and secondary analysis. The dataset was developed using a structured questionnaire grounded in the Unified Theory of Acceptance and Use of Technology (UTAUT2) framework together with additional sustainability and behavioural constructs, including EC, HB, PI, RS, and CCB. Responses were collected using a five-point Likert scale ranging from 1 = strongly disagree to 5 = strongly agree. The final dataset contains 60 variables, including demographic variables and construct measurement items. The dataset is organized into two major categories: demographic variables and latent construct measurement items. Demographic variables include age, education level, gender, occupation, and monthly income. The remaining variables represent multi-item constructs associated with hybrid car adoption behaviour and sustainability perceptions. The dataset also enables comparative analysis of green technology adoption behaviour in emerging economies. Fig. 1 Illustrates the overall dataset repository structure.

### 3.1 Respondent demographic characteristics

**Fig. 2:**
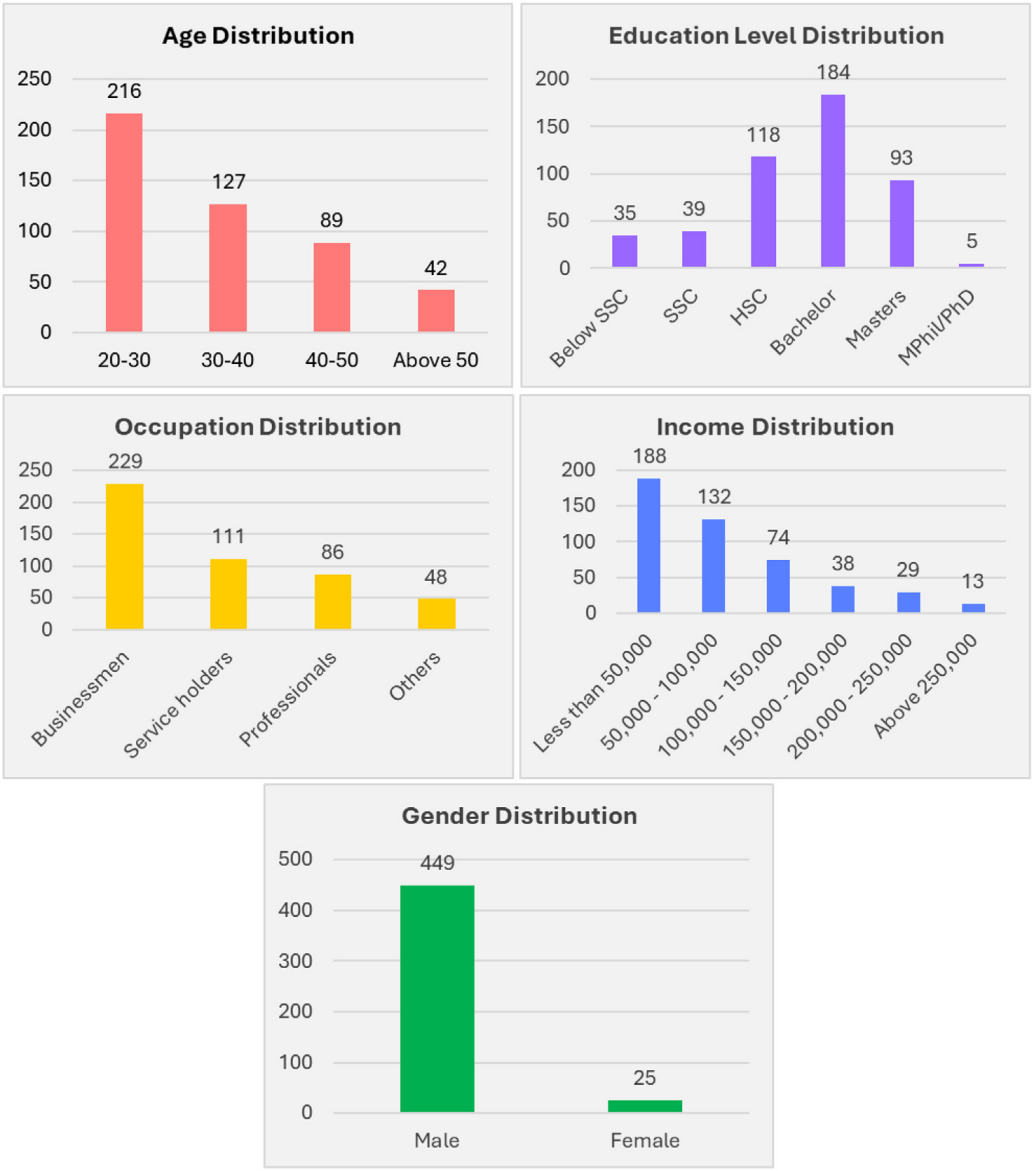
Demographic distribution of respondents across age, education, gender, occupation, and income categories. The figure illustrates the composition of the sample used for analyzing hybrid car adoption behaviour in Bangladesh.

The demographic profile of the respondents is summarized in Table 2 and is illustrated in Fig. 2. Among the 474 respondents, the majority were male participants (94.7%), while female respondents accounted for 5.3% of the total sample. In terms of age distribution, most respondents belonged to the 20–30 years age group (45.6%), followed by the 30–40 years category (26.8%). Respondents aged 40–50 years represented 18.8% of the sample, while individuals above 50 years accounted for 8.9%. Regarding educational qualification, the largest proportion of respondents held bachelor degrees (38.8%), followed by higher secondary certificate (HSC) holders (24.9%) and master’s degree holders (19.6%). A comparatively smaller proportion of respondents reported secondary-level education or below. This educational profile indicates that the survey predominantly captured opinions from relatively educated consumers familiar with technological and environmental issues. Occupation-wise, businesspersons constituted the largest respondent group (48.3%), followed by service holders (23.4%), professionals (18.1%), and respondents from other occupations (10.1%). Monthly income analysis revealed that the majority of respondents belonged to the lower and middle-income categories. Approximately 39.7% of respondents reported monthly income below BDT 50,000, while 27.8% reported income between BDT 50,000 and BDT 100,000. Higher income groups represented comparatively smaller portions of the sample. The demographic composition indicates that the dataset mainly reflects perceptions of economically active urban and semi-urban consumers with moderate to high educational exposure. Such respondents are comparatively more aware of sustainable transportation technologies and ECs associated with traditional fuel-based transportation systems.

**Table 2:** Demographic characteristics of respondents (*n* = 474).

Variable	Category	Frequency	Percentage (%)
Age	20–30 years	216	45.6
	30–40 years	127	26.8
	40–50 years	89	18.8
	Above 50 years	42	8.9
Education Level	Below SSC	35	7.4
	SSC	39	8.2
	HSC	118	24.9
	Bachelor	184	38.8
	Masters	93	19.6
	MPhil/PhD	5	1.1
Gender	Male	449	94.7
	Female	25	5.3
Occupation	Business	229	48.3
	Service	111	23.4
	Professional	86	18.1
	Others	48	10.1
Monthly Income (BDT)	<50,000	188	39.7
	50,000–100,000	132	27.8
	100,000–150,000	74	15.6
	150,000–200,000	38	8.0
	200,000–250,000	29	6.1
	Above 250,000	13	2.7

### 3.2 Dataset structure and variable composition

The dataset includes multiple constructs derived from technology acceptance and green consumer behaviour literature. The constructs include PE, EE, RS, FC, SI, HM, PV, HB, EC, PI, CCB, and BIP. Each construct contains multiple observed variables measured using Likert-scale responses. PE measures perceived usefulness and utility associated with hybrid cars. EE evaluates ease of learning and usability perceptions. RS examines the influence of salesperson behaviour and communication on purchase decisions. FC assess respondents’ perceived availability of resources, knowledge, and technological compatibility. SI captures the impact of family, peers, and social trends on hybrid car adoption. HM measures enjoyment and entertainment associated with hybrid car usage, while PV evaluates cost-benefit perceptions and affordability. HB and EC constructs capture sustainability-related perceptions concerning health consciousness and environmental awareness. PI measures respondents’ willingness to explore and adopt emerging technologies. CCB examines advocacy and recommendation behaviour, while behavioural intention reflects future adoption willingness and continuous usage intention. The dataset structure supports advanced behavioural modelling and allows researchers to investigate direct, indirect, and mediating relationships among sustainability perceptions, technology acceptance factors, and hybrid car adoption intention.

The descriptive statistics presented in Table 3 indicate moderate to relatively high respondent agreement across most constructs. HB recorded the highest mean value (*M* = 3.389), suggesting that respondents strongly associate hybrid cars with positive health-related outcomes and sustainable lifestyles. HM (*M* = 3.329), behavioural intention (*M* = 3.286), and EC (*M* = 3.269) also demonstrated relatively high agreement levels, indicating positive perceptions toward environmentally friendly transportation technologies. Conversely, RS (*M* = 2.884) and SI (*M* = 2.978) exhibited comparatively lower mean scores, suggesting that interpersonal persuasion and social pressure may have relatively weaker influence on hybrid car adoption compared with intrinsic sustainability and utility-related motivations. The standard deviation values across constructs ranged from 0.932 to 1.119, indicating moderate variability in responses and sufficient heterogeneity for advanced statistical analysis. The variability in responses further supports the applicability of multivariate analysis and predictive modelling approaches.

**Table 3:** Construct-wise dataset composition and descriptive statistics.

Construct	Number of Items	Mean	Standard Deviation	Minimum	Maximum
PE	5	3.114	0.946	1.0	5.0
EE	4	3.252	1.070	1.0	5.0
RS	5	2.884	0.963	1.0	5.0
FC	5	3.051	0.967	1.0	5.0
SI	5	2.978	1.011	1.0	5.0
HM	3	3.329	1.102	1.0	5.0
PV	4	3.292	1.091	1.0	5.0
HB	5	3.389	1.119	1.0	5.0
EC	4	3.269	1.036	1.0	5.0
PI	6	3.019	0.932	1.0	5.0
CCB	6	3.206	0.979	1.0	5.0
BIP	3	3.286	1.081	1.0	5.0

### 3.3 Measurement scale and coding scheme

All construct items were measured using a five-point Likert scale where:

• 1 = Strongly Disagree
• 2 = Disagree
• 3 = Neutral
• 4 = Agree
• 5 = Strongly Agree

The demographic variables were coded numerically to facilitate statistical analysis and structural equation modelling. The dataset was cleaned and preprocessed using spreadsheet software and statistical tools before import into SmartPLS for measurement and structural model analysis.

The dataset includes:

• Raw survey responses
• Variable dictionary
• Questionnaire instrument
• Construct-item mapping
• Numerical coding scheme

The coding structure enables direct reuse of the dataset in statistical software packages including SPSS, SmartPLS, R, Python, and STATA.

### 3.4 Geographic coverage and survey context

**Fig. 3:**
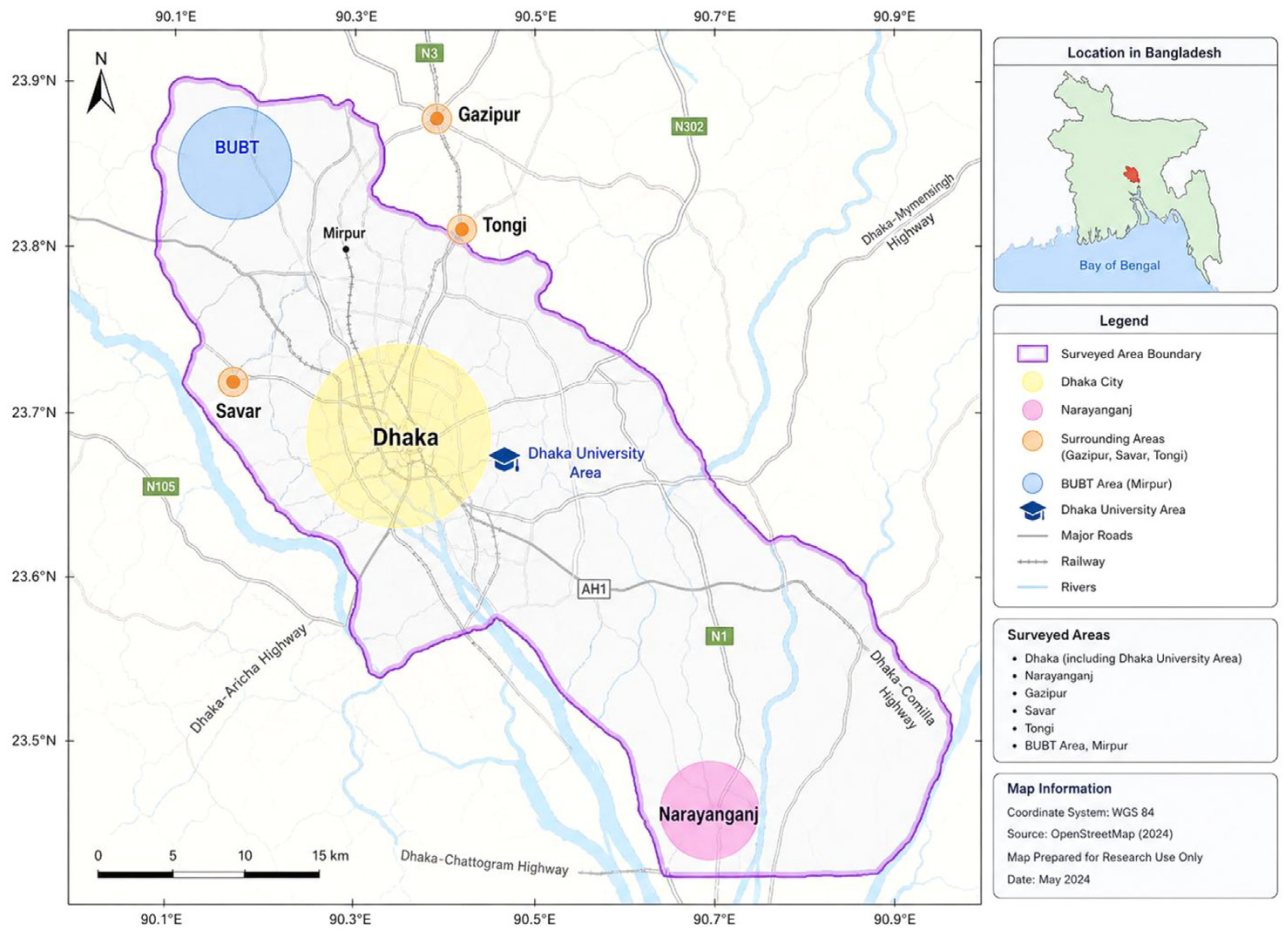
Geographic distribution of the surveyed respondents across Dhaka and surrounding urban and semi-urban areas of Bangladesh.

The survey was conducted primarily in urban and semi-urban regions of Bangladesh, including Dhaka and surrounding areas where awareness and accessibility of hybrid vehicle technologies are comparatively higher. These locations represent economically active regions with growing concerns regarding environmental pollution, fuel efficiency, and sustainable transportation. The geographic distribution of respondents is illustrated in Fig. 3. The surveyed regions were selected because they contain comparatively higher concentrations of private vehicle users, professionals, businesspersons, and consumers with exposure to emerging transportation technologies. The contextual setting of Bangladesh as a developing economy provides important insight into green technology adoption behaviour under resource-constrained market conditions. The dataset therefore contributes to the growing body of sustainable mobility research from emerging economies and provides opportunities for comparative analysis with developed and developing country contexts.

### 3.5 Potential reuse and analytical applicability

The dataset can be reused for various analytical and methodological purposes. Researchers may employ the data for structural equation modelling, machine learning prediction, clustering analysis, regression analysis, mediation analysis, and consumer segmentation studies. The availability of demographic variables further enables comparative subgroup analysis based on age, education, income, and occupation. The dataset also supports theoretical extension of UTAUT2 and green consumer behaviour frameworks in the context of sustainable transportation technologies. Policymakers may utilize the dataset to identify major barriers and drivers influencing hybrid car adoption in developing economies. Similarly, automobile marketers and sustainability practitioners can use the findings to formulate awareness campaigns, green mobility strategies, and targeted communication approaches for environmentally conscious consumers. Furthermore, the dataset may serve as a benchmark resource for future comparative studies on electric vehicles, sustainable transportation, and green technology acceptance across South Asian and emerging-market contexts.

Compared with recent demographic characteristics reported by the Bangladesh Bureau of Statistics (BBS) [11], the surveyed sample reflects a comparatively more urban, educated, and economically active respondent group. This distribution is consistent with the exploratory objective of examining hybrid vehicle adoption perceptions among consumers more likely to possess awareness, purchasing capacity, and exposure to sustainable transportation technologies. Therefore, the dataset should not be interpreted as nationally representative of the entire Bangladeshi population.

### 3.6 Reliability and validity assessment

The reliability and convergent validity of the measurement constructs were evaluated using Cronbach’s alpha, rho_A, Composite Reliability (CR), and average variance extracted (AVE) obtained from SmartPLS analysis. As presented in Table 4, all constructs demonstrated satisfactory internal consistency reliability because Cronbach’s alpha and composite reliability values exceeded the recommended threshold of 0.70. Similarly, all AVE values were greater than 0.50, confirming adequate convergent validity of the measurement model. The results indicate that the construct indicators consistently measured their corresponding latent variables and were statistically appropriate for subsequent structural equation modelling analysis.

**Table 4:** Reliability and Convergent Validity Assessment of Measurement Constructs.

Construct	Cronbach’s Alpha	rho_A	Composite Reliability (CR)	Average Variance Extracted (AVE)
BIP	0.934	0.939	0.958	0.884
CCB	0.941	0.944	0.953	0.773
EC	0.948	0.949	0.962	0.864
EE	0.932	0.938	0.952	0.831
FC	0.896	0.906	0.923	0.705
HB	0.951	0.952	0.963	0.837
HM	0.949	0.950	0.967	0.907
PE	0.869	0.886	0.905	0.658
PI	0.909	0.913	0.930	0.688
PV	0.922	0.933	0.944	0.809
RS	0.901	0.912	0.926	0.716
SI	0.914	0.914	0.937	0.748

## 4 Experimental Design, Materials and Methods

**Fig. 4:**
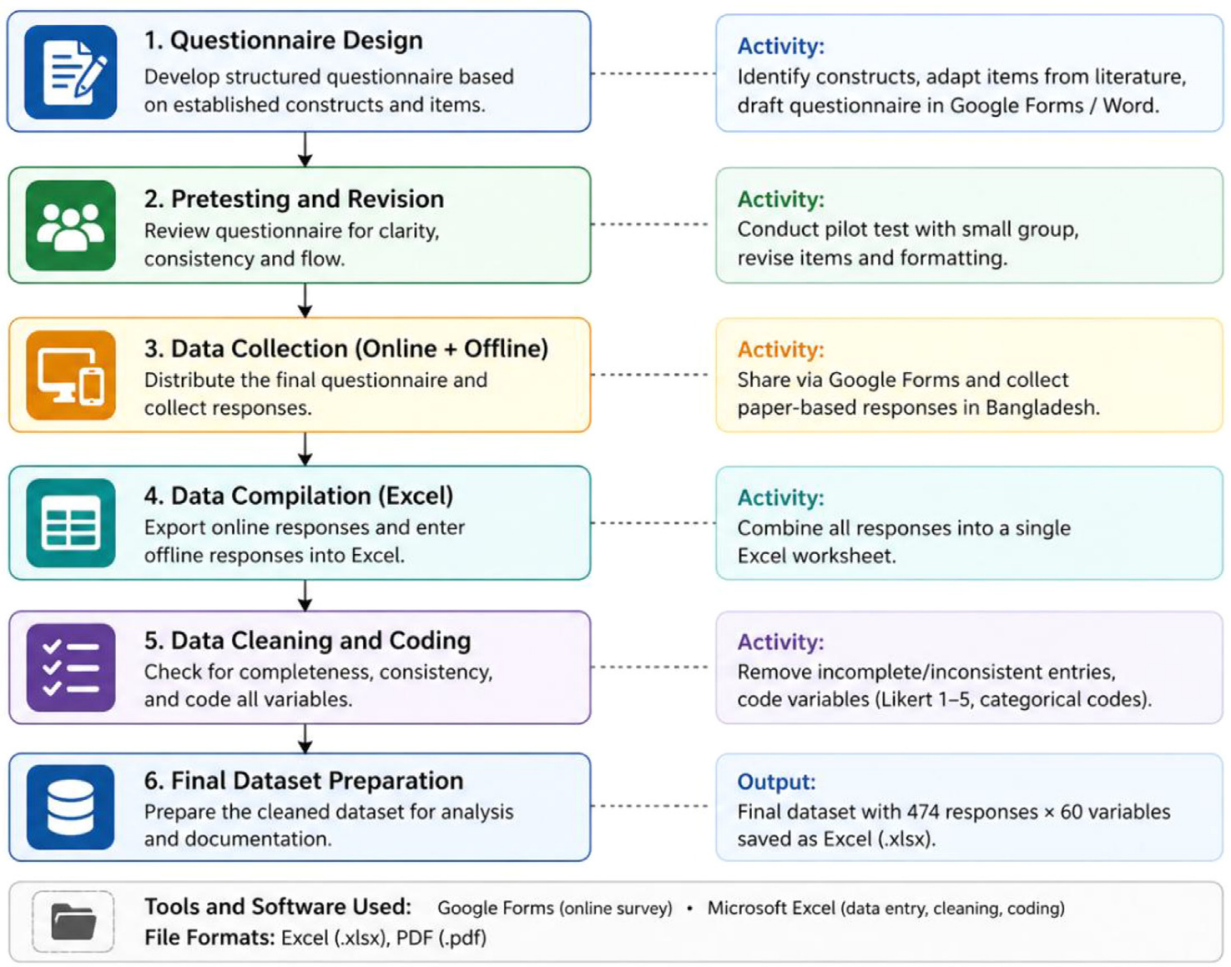
Data collection and pre-processing workflow illustrating the sequential stages from questionnaire design to final dataset preparation, including data collection, compilation, cleaning, and coding.

The dataset was generated through a structured survey designed to examine factors influencing hybrid car adoption behaviour and sustainability-related perceptions among consumers in Bangladesh. The questionnaire framework was primarily grounded in the Unified Theory of Acceptance and Use of Technology (UTAUT2) model together with additional sustainability-oriented behavioural constructs frequently applied in green technology adoption studies. The UTAUT2-related items (PE, EE, FC, SI, HM, PV, and BIP) were adapted from Tamilmani et al. [1], while sustainability-related constructs including EC, HB, PI, and CCB were adapted from prior green consumption and technology adoption studies [[2], [3], [4], [5], [6], [7], [8]]. The survey instrument incorporated validated measurement items adapted from prior literature relating to technology acceptance, EC, CCB, PI, and sustainable consumption behaviour. The complete questionnaire instrument is provided alongside the dataset repository for transparency and reproducibility. The questionnaire was administered in English because the target respondents were educated urban and semi-urban consumers familiar with English-language survey instruments. No Artificial Intelligence (AI)-based tools or automated questionnaire-generation systems were used during the development, adaptation, or validation of the survey instrument. The questionnaire consisted of two major sections. The first section collected demographic information including age, education level, gender, occupation, and monthly income. The second section measured latent constructs associated with hybrid car adoption behaviour, including PE, EE, RS, FC, SI, HM, PV, HB, EC, PI, CCB, and BIP. All measurement items were evaluated using a five-point Likert scale ranging from 1 = strongly disagree to 5 = strongly agree. The use of a five-point Likert scale was considered appropriate because it provides balanced response sensitivity while reducing respondent fatigue and improving interpretability in consumer behavioural research. The survey employed a modified questionnaire approach rather than a fully standardized instrument. Measurement items were adapted from previously validated scales and contextualized for hybrid car adoption behaviour in Bangladesh. Minor linguistic and contextual modifications were introduced to improve readability, respondent comprehension, and contextual relevance for consumers in an emerging economy setting. Prior to final data collection, the questionnaire was pretested among a small group of respondents to evaluate wording clarity, item consistency, and survey flow. Based on participant feedback, several statements were refined for improved comprehensibility and response consistency. Data were collected between March 2025 and July 2025 using both online and paper-based survey approaches. Respondents were approached through university networks, professional communities, social media platforms, automobile-related communities, and direct physical distribution in urban and semi-urban commercial areas. The survey primarily targeted respondents familiar with hybrid vehicles, sustainable transportation technologies, or environmentally friendly mobility solutions. Participation was voluntary and anonymous, and no financial incentives or rewards were provided to respondents. A non-probability convenience sampling strategy was adopted because of the exploratory nature of the study and the limited accessibility of hybrid car user populations in Bangladesh. A probability-based sampling approach was not feasible because no comprehensive sampling frame exists for consumers in Bangladesh who are aware of or interested in hybrid vehicle technologies. Hybrid vehicle awareness is dispersed across the general population, and no official registry or database is available to identify eligible respondents. Consequently, a purposive sampling strategy was employed to recruit respondents who met the study's eligibility criteria and possessed sufficient familiarity with hybrid vehicle concepts to provide meaningful responses. No commercial survey panels, quota-based recruitment systems, or third-party respondent marketplaces were used during data collection. Respondents were recruited directly through institutional, professional, and community-based outreach channels. Sampling protocols were implemented to improve response relevance and data quality. Respondents were approached through purposive outreach in locations and communities where awareness of hybrid vehicles was comparatively higher, including universities, business communities, urban commercial centers, and online automobile-related social media groups. Prior to participation, respondents were informed regarding the purpose of the study and eligibility requirements. Only completed questionnaires from respondents indicating familiarity with hybrid vehicles or sustainable transportation concepts were retained for final analysis. A total of 510 questionnaires were initially collected. During pre-processing, incomplete questionnaires, duplicate responses, and inconsistent response patterns were removed through manual screening and spreadsheet-based validation procedures. After data cleaning, 474 valid responses were retained from 510 collected questionnaires, representing a valid-response retention proportion of approximately 92.9% after exclusion of incomplete and inconsistent responses. Because the survey employed mixed online and paper-based convenience sampling approaches, the total number of individuals exposed to the survey invitation or recruitment message was not systematically recorded; therefore, a formal population-level response rate could not be calculated. To improve participation, reminder messages were periodically shared through social media groups, university communication channels, and professional networks during the data collection period. No monetary incentives, gifts, or compensation were provided to respondents for participation. Of the 510 collected questionnaires, 36 responses were excluded because of incompleteness or inconsistent response patterns, resulting in a valid response rate of 92.9%. Most excluded questionnaires were removed because of partially completed construct items, missing demographic information, or inconsistent response patterns identified during manual screening. Because participation was voluntary and self-administered, a formal item-level partial response rate was not separately calculated. Only fully completed and internally consistent questionnaires were retained for the final dataset. Because the survey used a convenience sampling strategy, the response rate should be interpreted as a completion-based indicator rather than a probability-based population response estimate. The final sample size of 474 valid responses was considered adequate for the exploratory and multivariate analytical objectives of this study. Although a formal statistical power analysis was not conducted, the sample size exceeds commonly recommended thresholds for Partial Least Squares Structural Equation Modelling (PLS-SEM) and behavioural research involving multiple latent constructs and indicators. Prior methodological studies have suggested that SEM-based analyses generally require sufficiently large samples relative to the number of construct indicators to ensure stable parameter estimation and reliable model evaluation. Therefore, the retained sample size was considered appropriate for descriptive analysis, construct reliability assessment, and structural modelling applications used in this dataset. The geographic distribution of the survey respondents is illustrated in Fig. 3. Data were collected primarily from Dhaka and surrounding urban and semi-urban regions where awareness, technological exposure, transportation demand, and purchasing capacity related to hybrid vehicles are comparatively higher. These locations were selected because they represent economically active areas characterized by increasing ECs, fuel price sensitivity, and growing interest in sustainable transportation alternatives. Fig. 4 presents the complete data collection and pre-processing workflow adopted in this study. The workflow includes questionnaire development, pilot testing, respondent selection, survey administration, response compilation, pre-processing, coding, and final dataset preparation. Paper-based responses were manually entered into spreadsheet format and cross-checked for consistency. Responses collected through online and paper-based modes were merged into a unified spreadsheet dataset using consistent variable coding structures and identical questionnaire item ordering. Manual cross-checking procedures were conducted after data entry to ensure consistency between online and offline response formats before final dataset consolidation. Missing values were handled through case-wise exclusion during the pre-processing stage. Questionnaires containing incomplete construct responses or substantial missing information were removed prior to final dataset preparation. No statistical imputation procedures were applied because only complete and internally consistent responses were retained for analysis. Potential outliers and inconsistent response patterns were manually screened using spreadsheet-based validation and descriptive statistical inspection before importing the dataset into SmartPLS. Construct-level descriptive statistics indicated moderate to positive respondent perceptions across most sustainability and technology acceptance dimensions. The dataset demonstrated sufficient variability and heterogeneity for advanced statistical modelling and predictive analysis.

No post-stratification weighting or calibration weighting procedures were applied because the dataset was generated using a non-probability convenience sampling approach rather than probability-based survey sampling. Consequently, all analyses were conducted using unweighted responses, and findings should be interpreted as exploratory representations of the surveyed sample rather than nationally representative population estimates. Partial Least Squares Structural Equation Modelling (PLS-SEM) using SmartPLS was considered appropriate for this dataset because the study aimed to examine complex relationships among multiple latent constructs associated with technology acceptance and sustainability perceptions. PLS-SEM is particularly suitable for exploratory behavioural research involving latent variables, prediction-oriented analysis, non-normal survey data, and relatively complex construct structures. The method additionally supports simultaneous evaluation of measurement reliability, convergent validity, and structural relationships among constructs within emerging theoretical contexts such as hybrid vehicle adoption behaviour in developing economies.

### 4.1 Data usage example

The python code example below demonstrates a simple workflow for loading the dataset and performing preliminary statistical exploration using Python and the pandas library. Researchers may further extend the dataset for structural equation modelling, regression analysis, clustering, predictive modelling, and machine learning applications using statistical software such as SPSS, SmartPLS, R, or Python.

**import pandas as pd.**

**# Load dataset.**

**df = pd.read_excel("Adoption_of_Hybrid_Cars_DATASET.xlsx").**

**# Display first five rows.**

**print(df.head()).**

**# Basic descriptive statistics.**

**print(df.describe()).**

**# Correlation matrix.**

**correlation_matrix = df.corr(numeric_only=True).**

**# Display correlation matrix.**

**print(correlation_matrix).**

## Limitations

The dataset is based on a non-probability convenience sampling approach, which may limit representativeness across the broader population. Because the dataset was collected using non-probability convenience sampling rather than probability-based random sampling, conventional sampling error and margin-of-error estimation procedures are not statistically appropriate. Therefore, the dataset should be interpreted as exploratory and analytical rather than nationally representative. The respondent distribution was predominantly male, which may limit gender-balanced interpretation of hybrid vehicle adoption behaviour. Data were collected primarily from urban and semi-urban respondents in Bangladesh, which may underrepresent rural perspectives. Because respondents were recruited primarily through university networks, professional communities, and automobile-related social media groups, the sample may overrepresent educated and technology-aware consumers. The use of a self-administered questionnaire introduces the possibility of response bias, including social desirability and subjective interpretation of items. All variables are measured using a 5-point Likert scale, which may limit variability in responses and capture of nuanced opinions. The dataset is cross-sectional, reflecting responses at a single point in time without temporal variation. Data entry for paper-based responses involved manual transcription into spreadsheet format, which may introduce minor input errors despite checking procedures. Additionally, only respondents with basic awareness of hybrid cars were included, which may exclude uninformed population segments. No objective behavioural or usage data were collected; all responses are based on self-reported perceptions and intentions. Despite increasing public awareness regarding sustainable transportation, several challenges continue to limit broader hybrid vehicle adoption in Bangladesh. These challenges include comparatively high upfront purchasing costs, limited supporting infrastructure and maintenance facilities, insufficient policy incentives, and restricted technological accessibility outside major urban regions. In addition, inadequate public awareness regarding long-term economic and environmental benefits may further constrain market expansion for hybrid vehicle technologies in developing economies. The dataset presented in this study may support future policy formulation and evidence-based sustainable transportation planning by enabling researchers and policymakers to identify major behavioural, economic, and technological drivers influencing hybrid vehicle adoption. Furthermore, the dataset provides opportunities for advanced market segmentation, consumer profiling, predictive modelling, and comparative sustainable mobility research across different demographic and socio-economic groups. The publicly accessible structure of the dataset may also facilitate future machine learning applications, behavioural modelling studies, and green transportation strategy development within emerging economy contexts.

## Ethics Statement

This study involved voluntary participation from adult respondents through an anonymous questionnaire survey. Informed consent was obtained from all participants prior to participation. Respondents were informed about the purpose of the study, the confidentiality of their responses, and their right to withdraw at any time without consequence. No personally identifiable information was collected, and all data were fully anonymized before analysis. The study employed a non-invasive, minimal-risk survey design and followed institutional research guidelines and the ethical principles of the Declaration of Helsinki. The study followed institutional research guidelines for anonymous minimal-risk social science survey research. Because no personally identifiable or sensitive information was collected and participation was voluntary and anonymous, formal ethical review board approval was not required .

## CRediT Author Statement

**Md. Aslam Uddin:** Conceptualization, Data curation, Formal analysis, Investigation, Methodology, Resources, Software, Supervision, Validation. **Mohammad Zahedul Alam:** Data curation, Formal analysis. **Md. Masudul Islam:** Visualization, Writing – original draft

## Data Availability

Mendeley DataHybrid Car Adoption and Consumer Behavior Dataset (Dhaka) (Original data)
